# Transcriptome Analysis of the Developmental Effects of Bisphenol F Exposure in Chinese Medaka (*Oryzias sinensis*)

**DOI:** 10.3390/ijms241310898

**Published:** 2023-06-30

**Authors:** Zhiying Liang, Yafen Guo, Duan Pi, Xiang Li, Bingying Li, Yongsi Huang, Xiaohong Song, Ramji Kumar Bhandari, Xuegeng Wang

**Affiliations:** 1 Institute of Modern Aquaculture Science and Engineering, Guangdong-Macao Joint Laboratory for Aquaculture Breeding Development and Innovation, College of Life Sciences, South China Normal University, Guangzhou 510631, China; 2 The Guangxi Key Laboratory of Theory and Technology for Environmental Pollution Control, Guilin University of Technology, Guilin 541000, China; 3 Department of Biology, University of North Carolina Greensboro, Greensboro, NC 27412, USA

**Keywords:** Bisphenol F, Chinese medaka, toxicity, developmental defects, endocrine disruptor

## Abstract

Bisphenol F (BPF) has been used in the syntheses of polymers, which are widely used in coatings, varnishes, adhesives, and other plastics. During the past decades, BPF contamination in the aquatic environment has dramatically increased due to its release from manmade products. Concerns have driven much attention to whether it may adversely impact aquatic lives or human beings. The present study performed an acute toxic exposure experiment and a 15 d developmental exposure of BPF at environmental concentrations (20, 200, and 2000 ng/L) using Chinese medaka (*Oryzias sinensis*). In the acute toxic exposure, the LC_50_ of BPF to Chinese medaka is 87.90 mg/L at 96 h. Developmental exposure induced a significant increase in the frequency of larvae with abnormalities in the 2000 ng/L BPF group compared to the control group. Transcriptomic analysis of the whole larvae revealed 565 up-regulated and 493 down-regulated genes in the 2000 ng/L BPF exposure group. Analysis of gene ontology and KEGG pathways enrichments indicated endocrine disorders to be associated with BPF-induced developmental toxicity. The present results suggest that BPF is developmentally toxic at 2000 ng/L concentration in Chinese medaka and causes endocrine-related aberrations in the transcriptional network of genes.

## 1. Introduction

Bisphenol F (CAS: 620-92-8, 4,4′-Dihydroxydiphenylmethane), which has high structural similarity to bisphenol A (BPA), is widely used in coatings, varnishes, linings, adhesives, and other plastics [1]. Bisphenol A, a well-studied endocrine-disrupting chemical (EDC), has been demonstrated to cause adverse effects in reproduction [2,3], prostate development [4], induction of obesity [5], and impairment of spermatogonial stem cell function [6]. Many countries strictly restrict the production and use of BPA. As a result, bisphenol F (BPF) has been gradually used in various consumer products as a substitute for BPA [7]. With the growing large-scale production and use, BPF has been frequently detected in most environmental samples and human biological samples.

Many studies have measured various bisphenol compounds in environmental samples and concluded that the BPF could be detected in most surface waters worldwide with increasing concentrations, suggesting it to be an emerging global pollutant [8,9,10,11]. In 2002, Fromme et al. reported that BPF was detectable in 77% of the surface-water samples, 72% of the sewage water samples, 58% of the sediment samples, and 87% of the sewage sludge samples, at significantly lower levels than BPA in Germany [12]. While in 2015, Yamazaki et al. measured the concentrations of eight bisphenol analogues in surface water collected from rivers and lakes in Japan, South Korea, China, and India [11]. They found that the BPF has higher concentrations than BPA in parts of those locations, such as the Pearl River in China and the Cooum River and Adyar River in India [11]. 

The BPF was detectable in 87% of water samples collected from Taihu Lake in September 2013, with a mean concentration of 0.83 ng/L [9]. However, in another set of water samples collected in November 2016 from Taihu Lake, the detection frequency increased to 100%, and the mean concentration increased to 78 ng/L [10]. By comparing the two studies, it can be concluded that the concentration of BPF in Taihu Lake increased dramatically just in three years.

Bisphenols (BPs) have been detected in sewage and wastewater effluent as well. Sun et al. collected sewage samples from seven sewage treatment plants in Xiamen, and found that the average concentration of BPF in inlet water was 50.0 ng/L, second only to BPA; and the BPF removal rate of the sewage treatment plants was about 93.8% [13]. Another study in the Dalian sewage treatment plant found that BPF with an inlet concentration of 66.4 ng/L was almost completely removed [14]. In 2015, one study reported that the BPF in effluent water is 3.39 ng/L in Europe [15]. Another study using the samples collected in effluent from sewage treatment plants in Slovenia and Croatia in 2017 found the BPF concentration to be 44.3 ng/L [16].

There are also studies on BPs in drinking water. Though the content of BPF in drinking water is generally lower than that in surface water, it is also frequently detected. Zhang et al. examined 16 BPs in raw water and drinking water collected from 20 drinking water treatment plants in China in 2017 [17]. The BPF content in raw water was ND-12.6 ng/L, accounting for 35%, while being detected in only one drinking water sample, accounting for 5% [17]. In a study conducted in Korea, BPF was detectable in 91% of tap water samples, 88% of purified water samples, and 89% of bottled water samples [18]. Meanwhile, BPF was detected in personal care products such as body lotion, shampoo, and soap, with a detectable rate between 2.6% to 13.4% in the United States and China [19]. BPF was frequently detected in plastic materials and paper products. 

As BPF is widely distributed in the environment, the increasing concerns about its potential adverse effects on human health and the environment require more in-depth study. Recent studies demonstrated that BPF has multiple toxic effects on organisms. It showed an endocrine-disrupting activity both in vivo and in vitro. Studies have shown that the BPF has estrogenic activity [20], and induced estrogen response in transgenic zebrafish [21], developmental neurotoxicity [22,23], and genetic toxicity [24]. In a comet assay, HepG2 cells showed DNA damage after 24 h exposure to BPF [24]. BPF could alter the transcription of genes involved in the HPT axis and cause thyroid endocrine disruption in zebrafish larvae and *Pelophylax nigromaculatus* tadpoles [25,26]. Long-term bisphenol F exposure induced different metabolic effects in the liver, intestinal microbiome composition, reproductive defects, and neurodevelopmental disorders in zebrafish and rats [27,28,29,30]. In contrast, many studies majorly focused on the concentration from μg/L to mg/L levels, which are higher than the actual concentration in the environment. The concern on how to evaluate the actual adverse effects realistically requires more investigations using aquatic animal models, such as fish, at environmental concentrations.

The Japanese medaka (*Oryzias latipes*), particularly the Hd-rR strain, is widely used in toxicological studies in laboratories worldwide [31]. It has several advantages, such as in vitro fertilization, transparent embryonic development, low cost, closer relationship to humans [32,33,34], genetic sex determination [35], and smaller genome size than zebrafish [36]. However, Japanese medaka and zebrafish do not inhabit in China and could not be faithfully used in toxicity studies in actual aquatic environments. The present study utilized an emerging new animal model, Chinese medaka (*Oryzias sinensis*) [37], to explore the toxicity of BPF. Chinese medaka has multiple advantages similar to the Japanese medaka, such as small body size, short generation time, genetic sexual determination, and well-annotated genomic information [38]. Furthermore, it is distributed in most parts of East Asia, and is convenient and suitable for both laboratory and field investigations [39]. The present study aims to examine the acute toxicity, developmental defects, and transcriptional alterations induced by BPF exposures in Chinese medaka embryos and provide information for understanding the health effects and ecological risk evaluation of BPF.

## 2. Results

### 2.1. Acute Toxicity of BPF to Chinese Medaka Embryo

**Figure 1:**
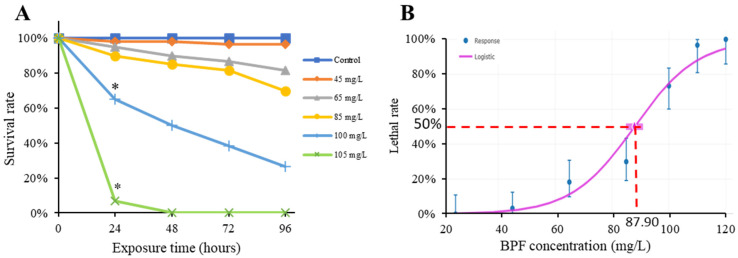
Results of the acute toxic experiment. (**A**): Survival rates of Chinese medaka embryos exposed to 0 (Control), 45, 65, 85, 100, 105 mg/L BPF within 96 h; (**B**): Lethality rates of Chinese medaka embryos exposed to BPF at 96 h. Dot line showed the 96 h LC_50_ is 87.90 mg/L. Asterisks indicate statistical significance (* *p* < 0.05).

In a 96-h acute toxicity experiment, the survival rates gradually decreased with the exposure concentration increased (Figure 1A). Using one-way ANOVA, the survival rates were found to be significantly decreased in 100 mg/L and 105 mg/L treatment groups, beginning from 24 h (Figure 1A). According to the lethal rates at 96 h, a fitting curve was generated to calculate the LC_50_. As shown in Figure 1B, the 96 h LC_50_ for BPF on Chinese medaka embryos is 87.90 mg/L.

### 2.2. Developmental Abnormalities Induced by BPF Exposure

**Figure 2:**
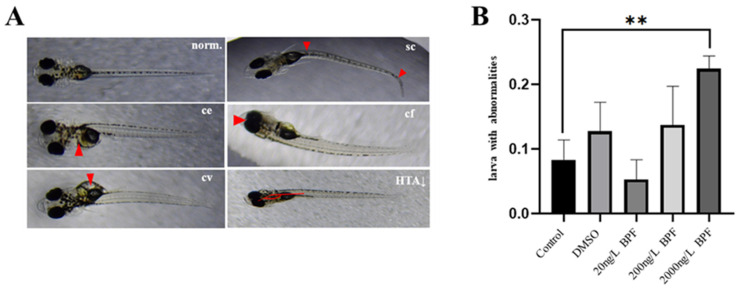
Developmental abnormalities induced by BPF exposure at 15 dpf. (**A**) Normal fry (norm.), fry with pericardial edema (ce), fry with enlarged yolk sac (cv), fry with spinal curvature (sc), fry with craniofacial abnormalities (cf), fry with decreased head-trunk angle (HTA↓); (**B**): the frequency of larvae with abnormalities. Data represent as Mean ± SEM. Asterisks indicate statistical significance (** *p* < 0.01).

To investigate the adverse effects of BPF exposure on the development of Chinese medaka, embryos were exposed to 0, 20, 200, 2000 ng/L BPF and solvent control from 0 to 15 dpf. The growth parameters were recorded and analyzed. Results showed that there were no significant changes in survival rates, body length, or heart beats (Figure S1A–C). Developmental abnormalities induced by BPF exposure were analyzed, including pericardial edema, enlarged yolk sac, craniofacial abnormality, curvature of spine, and decreased head-trunk angle (Figure 2A). No significant differences were found in those abnormalities when individual teratogenic effect was considered (Figure S2A–E). However, the frequency of larvae with all type of abnormalities had a significant increase in the 2000 ng/L group (*t*-Test, *p* < 0.01), demonstrating that BPF at higher environmental concentration can induce health defects in Chinses medaka (Figure 2B).

### 2.3. Differential Expression of Genes (DEGs) in the Larvae

To further investigate the mechanisms of the adverse effects of developmental BPF exposure on Chinese medaka, their transcriptome profile was determined by bulk RNA sequencing. After quality control and reads filtering, 39,504,122 to 58,837,952 high quality clean sequence reads were generated for each sample, and reads uniquely mapping ratio ranged from 70.43% to 76.21% (Table S1).

**Figure 3:**
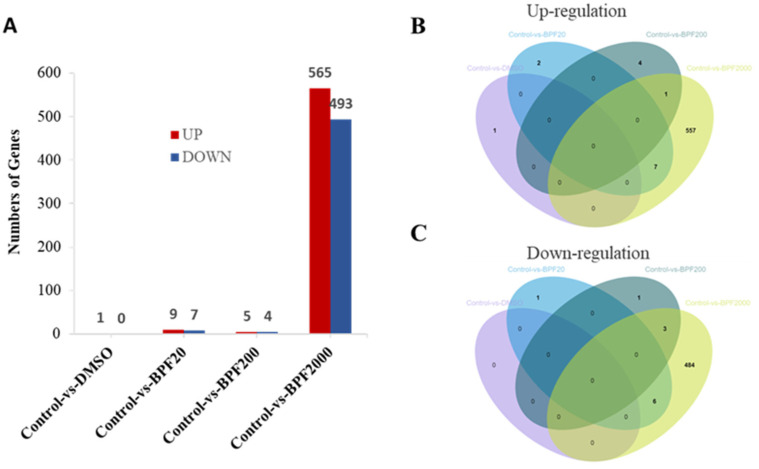
Differentially expressed genes(DEGs) statistics. (**A**): The numbers of DEGs in bisphenol F treatment groups compared to the control group; (**B**): Venn diagram of shared up-regulated DEGs among each group; (**C**): Venn diagram of shared down-regulated DEGs among each group.

Significant DEGs were identified in 20, 200, and 2000 ng/L BPF exposure groups compared to the control group. In brief, 16, 9, and 1058 DEGs were obtained when comparisons were made between 20 ng/L BPF (BPF20), 200 ng/L BPF (BPF200), 2000 ng/L BPF (BPF2000) versus control groups, respectively (Table S2). Among these DEGs, there were 9 up-regulated and 7 down-regulated genes in Control vs BPF20, 5 up-regulated and 4 down-regulated genes in Control vs BPF200, and 565 up-regulated and 493 down-regulated genes in Control vs BPF2000 (Figure 3A). These results indicated that the 2000 ng/L BPF can induce larger damage at the molecular level than at lower concentrations.

As shown in the Figure 3B,C, seven shared genes were both up-regulated in Control vs BPF20 and Control vs BPF2000 groups (Figure 3B), while three shared genes were both down-regulated (Figure 3C). One gene was up-regulated both in Control vs BPF200 and BPF2000 (Figure 3B), while three genes were down-regulated (Figure 3C). 

### 2.4. Analysis of Gene Ontology

To further analyze the mechanisms underlying BPF-induced teratogenic effects, gene ontology enrichment was performed. There were no (gene ontology) GO terms enriched in the 16 DEGs identified between control and BPF20. The nine DEGs identified between Control and BPF200 were enriched in Cellular Component and Molecular Function classes (Table S3). The 1058 DEGs identified between Control and BPF2000 were enriched in Biological Process, Cellular Component and Molecular Function classes (Table S4).

**Figure 4:**
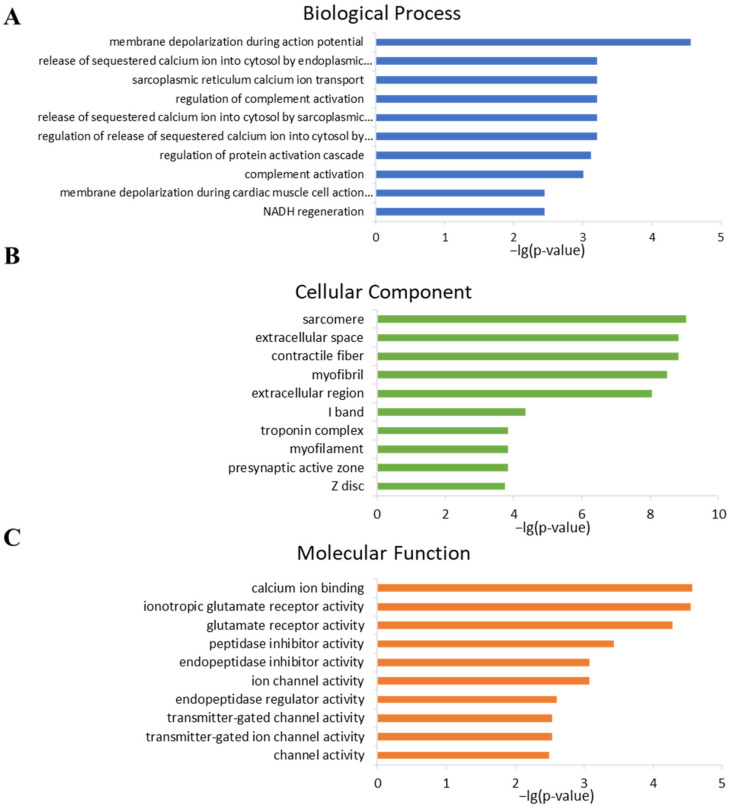
Gene Ontology (GO) enrichment of DEGs in 2000 ng/L BPF group compared to the Control group. (**A**): Top 10 GO enrichment terms in Biological process; (**B**): Top 10 GO enrichment terms in Cellular component; (**C**): Top 10 GO enrichment terms in Molecular function.

The top 10 terms in each GO class of the BPF2000 group are shown in Figure 4. In the biological progress, these DEGs enriched in “membrane depolarization during action potential”, “release of sequestered calcium ion”, “sarcoplasmic reticulum calcium ion transport”, “regulation of protein activation cascade”, “complement activation”, and “NADH regeneration” items (Figure 4A). In the cellular component, these DEGs enriched in “sarcomere”, “extracellular space”, “contractile fiber”, “myofibril”, “I band”, “troponin complex”, “myofilament”, “presynaptic active zone”, “Z disc” items (Figure 4B). In the molecular functions, these DEGs enriched in “calcium ion binding”, “glutamate receptor activity”, “peptidase inhibitor activity”, “endopeptidase inhibitor activity”, “ion channel activity”, “endopeptidase regulator activity”, “transmitter-gated ion channel activity” items (Figure 4C).

**Figure 5:**
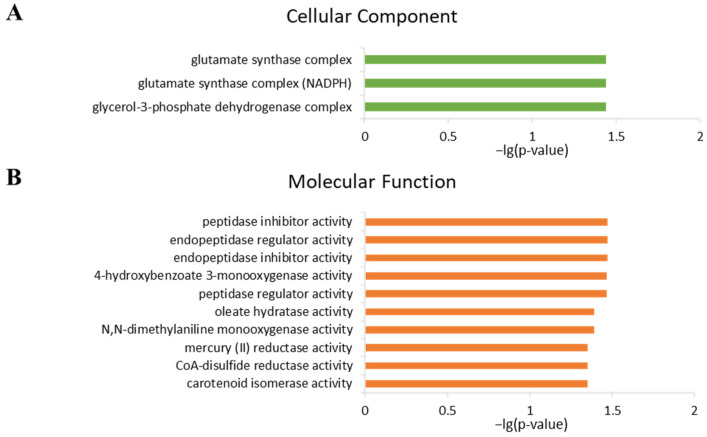
Gene Ontology (GO) enrichment of DEGs in 200 ng/L BPF group compared to the Control group. (**A**): Cellular component; (**B**): Top 10 GO enrichment terms in Molecular function.

The top 10 terms in each GO class of BPF200 group are shown in Figure 5. In the cellular components, these DEGs enriched in “glycerol-3-phosphate dehydrogenase complex”, “glutamate synthase complex (NADPH)” and “glutamate synthase complex” items (Figure 5A). In the molecular functions, these DEGs enriched in “peptidase inhibitor activity”, “endopeptidase regulator activity”, “endopeptidase inhibitor activity”, “peptidase regulator activity”, “oleate hydratase activity”, “N,N-dimethylaniline monooxygenase activity”, “carotenoid isomerase activity” items (Figure 5B). 

### 2.5. Analysis of Key KEGG Pathways

To further analyze the mechanisms underlying BPF-induced toxic effects in the larvae, Kyoto Encyclopedia of Genes and Genomes (KEGG) pathway enrichment was performed. There were no GO terms enriched in the 16 DEGs identified between control and BPF20. The KEGG items enriched with the nine DEGs identified between control and BPF200 have been listed in Table S5. The KEGG items enriched of the 1058 DEGs identified between Control and BPF2000 are shown in the Table S6. 

**Figure 6:**
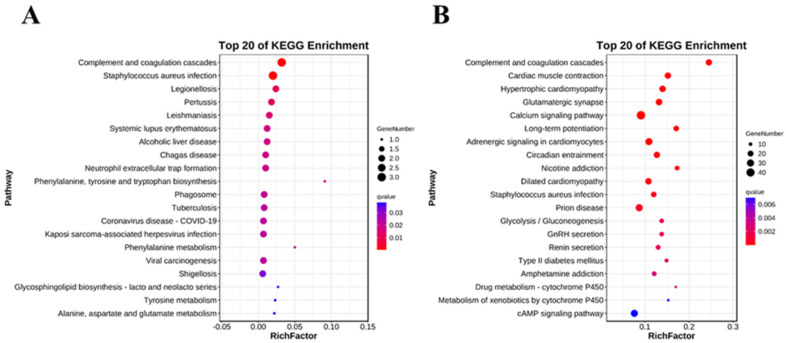
KEGG pathway enrichment of DEGs at different groups. (**A**): Top 20 KEGG enrichment pathways in 200 ng/L BPF group compared to Control group; (**B**): Top 20 KEGG enrichment pathways 2000 ng/L BPF group compared to Control group.

The top 20 pathways are shown in Figure 6. In the BPF200 group, the DEGs were enriched in “complement and coagulation cascades”, “staphylococcus aureus infection”, “legionellosis”, “pertussis”, “alcoholic liver disease”, “neutrophil extracellular trap formation”, and “phenylalanine, tyrosine and tryptophan biosynthesis” (Figure 6A). In the BPF2000 group, the DEGs were enriched in “complement and coagulation cascade”, “Cardiac muscle contraction”, “hypertrophic cardiomyopathy”, “glutamatergic synapse”, “calcium signaling pathway”, “long-term potentiation”, “adrenergic signaling in cardiomyocytes”, “circadian entrainment”, “nicotine addiction”, ‘dilated cardiomyopathy”, and “staphylococcus aureus infection” (Figure 6B).

The “complement and coagulation cascades” (Figure S3) and “Staphylococcus aureus infection” (Figure S4) pathways were highly enriched in both groups, indicating that these pathways may involve in the mechanism of BPF-induced adverse effects. Above all, the primary response pathways were immune system, circulatory system, nervous system and infectious disease. Complement and coagulation cascades is the main response pathway in the immune system pathway, indicating a close relationship between the toxicity mechanism of BPF and the balance of coagulation and fibrinolysis system as well as the biosynthesis of complements.

## 3. Discussion

In the present study, both acute and developmental toxicity of BPF were assessed using Chinese medaka as an animal model. The 96-h acute toxic exposure showed that BPF’s LC_50_ was 87.90 mg/L for Chinese medaka. A previous study had conducted the acute toxic experiments of six chemicals on Chinese medaka and found that the 96-h median lethal concentrations of Hg2þ, Cr6þ, linear alkylbenzene sulfonates, triclosan, 3,4-dchloroaniline, sodium chloride to *O. sinensis* were 0.29, 50, 6.0, 0.63, 9.2, and 14,400 mg/L, respectively. The present study found that after 96 h of acute toxic exposure to BPF, the half-lethal concentration of Chinese medaka is lower than that of Hg2þ, Cr6þ, linear alkylbenzene sulfonate, triclosan, and 3,4-dichloroaniline [37].

Li et al. exposed Rana embryos and tadpoles for 96 h with a series of concentrations of BPF, and concluded that the 96h-LC_50_ of BPF for Rana embryos, tadpoles, zebrafish embryos and adult zebrafish were 7.99 mg/L, 9.52 mg/L, 7.40 mg/L, and 9.51 mg/L [40]. Gao et al. found that the 96h-LC_50_ of BPF for zebrafish embryos was 24.50 mg/L [41], whereas Yang et al. found that the same outcome with zebrafish embryos was observed with 9.391mg/L [7]. Compared with other experimental animals above, Chinese medaka seems less sensitive to BPF. According to the chemical hazard assessment guidelines (HJ/T 154-2004), BPF was highly toxic to black-spotted frog embryos, tadpoles, zebrafish embryos, and zebrafish adults, while BPF is moderate toxic to Chinese medaka embryos. 

In the present study, the defect rate between the control group and the 2000 ng/L BPF exposure group showed that the frequency of larvae with abnormalities was significantly increased. This was consistent with the results that the number of DEGs in the 2000 ng/L BPF exposure group was the highest. To our knowledge, the highest BPF concentration reported in surface water is 2800 ng/L in Japan [11], which is much higher than the concentration examined in the present study, suggesting high ecological risk in the environment. In China, the BPF concentrations were also reported as high as 1600 ng/L in Taihu Lake [42], and 1320 ng/L in Qinhuai River [43]. However, the mean BPF concentration dramatically increased from 0.83 ng/L in September 2013 [9] to 78 ng/L in November 2016 [10] in Taihu Lake. The BPF concentrations in Taihu Lake probably have increased higher than 2000 ng/L, which is reported harmful in the present study, and calls more attention to the effects on aquatic organisms and ecology.

Previous literature reported the reproductive toxicity and teratogenicity of fluorene-9-bisphenol (BHPF), another popular BPA substitute, on Chinese medaka to explore its toxic mechanism [39]. They found that compared with the control group, there were 814, 1160, 1011, 1442, and 1031 DEGs identified in the fish of 0.01, 0.1, 1, and 10 μg/L BHPF groups by RNA-seq approach [39]. In our study, compared to the control group, the DEGs of BPF in 20, 200, 2000 ng/L (i.e., 0.02, 0.2, 2 μg/L) were 16, 9, and 1058, respectively. The comparison of 0.01 to 0.1 μg/L BHPF and 0.02, 0.2 μg/L BPF shows that in the same animal model, the toxicity of BPF is lower than that of BHPF at relatively low concentrations.

Intriguingly, several oxidase activities related GO terms were found enriched in the BPF200 group but not in the BPF2000 group, indicating that BPF employs different molecular mechanisms in toxicity at different concentrations. In cells, reactive oxygen species(ROS) detoxify through an antioxidant defense mechanism composed of free radical scavenger and specific antioxidant enzymes. However, excess ROS can escape antioxidant defenses and cause oxidative stress, leading to macromolecular oxidative damage and changes in cellular REDOX homeostasis [44]. Under environmental pressure, the production of free radicals will increase dramatically, resulting in oxidative stress on cells. The oxidative stress can oxidize the DNA, proteins, lipids, resulting in DNA damage, protein denaturation, loss of enzyme activity, and biofilm damage. Enzymatic and non-enzymatic antioxidants can constitute an essential biological defense against environmental pro-oxidants by countering the effects of ROS [45]. Qiu et al. reflected the antioxidant activity of the body through the changes of T-AOC, CAT, SOD, and MDA reflecting the degree of lipid peroxidation in zebrafish. They found that the content of reactive oxygen in zebrafish increased with high BPF exposure, resulting in oxidative compression, and the embryo or juvenile fish may produce toxic metabolites under oxidative stress [46].

In this study, KEGG pathways enrichment indicated “Staphylococcus aureus infection” pathways were highly enriched in both groups (Figure S4). Zhang et al. found most cell apoptotic processes caused by *Staphylococcus aureus* arise from the different kinds of secreted toxins such as *S. aureus* toxins, α-toxin, PVL and SEs [47]. In our study, the Staphylococcus aureus infection pathway is highly expressed in 200 ng/L BPF and 2000 ng/L BPF, which may cause cell apoptosis, leading to the toxic effect of BPF. Apoptosis is an important regulatory factor for cell growth and development. Studies have shown that BPA and its analogs significantly increase the apoptosis of larval brain and pericardial cells, and its toxicity to zebrafish embryos or larvae may be caused by developmental abnormalities caused by inducing cell apoptosis and disrupting cell homeostasis [48].

Another critical enriched pathway in 2000 ng/L BPF group is the “Metabolism of xenobiotics by cytochrome P450 pathway” (Table S6 and Figure S5). All nine involved DEGs are down-regulated in BPF exposure, indicating that the BPF may harm the ability of Chinese medaka to deal with the xenobiotics. Intriguingly, the “GnRH secretion” pathway was enriched in in 2000 ng/L BPF group too, and most of the involved DEGs are up-regulated (Table S6 and Figure S6). GnRH mediates the reproductive axis by a cascade of events involving pituitary GnRH receptors, release of gonadotropins by the pituitary, and secretion of sex steroids by gonads [49]. Up-regulation of genes related to GnRH secretion indicated that BPF exposure may have adverse effects on reproductive activities in Chinese medaka, which requires further investigation.

## 4. Materials and Methods

### 4.1. Fish Husbandry

The wild-type Chinese medaka used in this study was obtained from a local fish store, and cultured in the laboratory under standard conditions, with a temperature of 26.8 °C and a light/dark cycle of 14/10 h. The fish was confirmed by the COI gene sequence with the primers COI-F: ACCCTATATTTAATCTTCGGTGCTT and COI-R: GAACAGGTGCTGGTAGAGAATG. 

The animal protocols were approved by the Animal Care and Use Committee of the South China Normal University (No. SCNU-SLS-2022-025). All applicable institutional and/or national guidelines for the care and use of animals were followed. The fish were fed with brine shrimp and pellet feed three times a day. The eggs were collected regularly every morning, and the fertilized eggs with normal development were selected under the microscope for embryo developmental toxicity tests. 

### 4.2. Chemicals and Reagents

BPF (CAS:620-92-8, purity 99%) was purchased from Aladdin (Shanghai, China). Dimethyl sulfoxide (CAS:67-68-5, purity 99.9%) was purchased from Sigma-Aldrich (St. Louis, MO, USA).

### 4.3. Exposure Methods

Domesticated under laboratory conditions for two weeks, fish were kept in pre-aerated 24 h dechlorinated water with a pH of 7.8 ± 0.2. The water temperature was controlled at 26 ± 0.5 °C, and the photoperiod was 14 h light/10 h dark. Fish were fed freshly hatched brine shrimp once a day and dry feeds twice a day. Eggs were collected, separated, and cleaned with 3 g/L saline water about 1 h after feeding in the morning. Then, eggs were put in fresh salted water. Until the embryonic development reaches 6–7 hpf (hours post fertilization), eggs are transferred to a 6-well tissue culture plate for exposure. Experiments were conducted following the guiding principles of OECD (No.212, 210) [50,51].

### 4.4. Acute Toxic Exposure

At least three replicates were set in each treatment group. Ten zygotes were exposed in each hole for 96 h, during which the exposure solutions were replaced every 24 h. The stock solution of BPF was made by dissolving it in DMSO, then diluted into a working solution. The stock solutions of BPF were stored in the refrigerator at 4 °C away from light for not longer than 10 days. Each well, respectively, contains 3 g/L saline as a blank control group, 0.01% DMSO solution as a vehicle control group, 25, 45, 65, 85, 105, 110 and 120 mg/L BPF. The survival rate of the embryo was recorded every 24 h until 96 h with a microscope, and the median lethal concentration was calculated at 96 h (LC_50_). We used benchmark dose modelling to acquire the LC_50_ with the Benchmark Dose Software (BMDS) Online (https://bmdsonline.epa.gov/ (accessed on 26 June 2023). The median lethal concentration was calculated by fitting the exposure concentration–mortality curve with the logistic modeling under a benchmark response (BMR) 50%.

### 4.5. Developmental Toxic Exposure

At least 6 replicates were set and there were 10 zygotes in each well exposed for 15 dpf (day post fertilization), during which the exposure liquid was replaced every 48 h. The stock solution of BPF was made by dissolving it in DMSO, then diluted into a working solution. The stock solutions of BPF were stored in the refrigerator at 4 °C away from light for not longer than one month. Each well, respectively, contains water as a blank control group, 0.0001% DMSO solution as a vehicle control group, 20 ng/L BPF, 200 ng/L BPF, and 2000 ng/L BPF.

Microscopy was used daily until 15 dpf, and the survival rate, hatching rate, heart beating rate, blood circulation, and pigment were examined. At 15 dpf, the body length and heart beats of each larva and the proportion of various phenotypic changes were recorded. Phenotypic changes included pericardial edema, enlarged yolk sac, craniofacial abnormalities, changes in the size or shape of the jaw or eyes, spinal curvature, and decreased head-trunk angle. After the measurements, the samples were fixed using a Trizol reagent (Invitrogen, Carlsbad, CA, USA).

### 4.6. RNA-Seq and Bioinformatics Analysis

#### 4.6.1. Library Preparation and Sequencing

Total RNA was extracted using a Trizol reagent according to the manufacturer’s protocol. RNA quality was assessed on an Agilent 2100 Bioanalyzer (Agilent Technologies, Palo Alto, CA, USA) and checked using RNase-free agarose gel electrophoresis. After total RNA was extracted, mRNA was enriched by Oligo (dT) beads. Then, the enriched mRNA was fragmented into short fragments using a fragmentation buffer and reverse transcripted into cDNA with random primers. Second-strand cDNA was synthesized by DNA Polymerase I. Then, the cDNA fragments were purified with QIAquick PCR Extraction Kit (Qiagen, Venlo, The Netherlands), end-repaired, A-added, and ligated to Illumina sequencing adapters. The ligation products were size-selected by agarose gel electrophoresis, PCR amplified, and sequenced using Illumina Novaseq6000.

#### 4.6.2. Data Processing

Reads obtained from the sequencing were further filtered by FASTQ (version 0.18.0) [52]. Each sample’s mapped reads were assembled using StringTie (v1.3.1) [53,54] in a reference-based approach. For each transcription region, an FPKM (fragment per kilobase of transcript per million mapped reads) value was calculated to quantify its expression abundance and variations, using RSEM [55] software. Therefore, the calculated gene expression can be directly used to compare the gene expression differences among samples. Differentially expressed genes (DEGs) identification was performed by DESeq2 [56] software between two different groups. The genes/transcripts with the parameters of false discovery rate (FDR) below 0.05 and absolute fold change ≥ 2 were considered differentially expressed.

#### 4.6.3. GO Enrichment Analysis

Gene ontology (GO) [57] is an international standardized gene functional classification system that offers a dynamic-updated controlled vocabulary and a strictly defined concept to comprehensively describe the properties of genes and their products in any organism. Firstly, all DEGs were mapped to GO terms in the gene ontology database (http://www.geneontology.org/ (accessed on 2 February 2023), gene numbers were calculated for every term, and significantly enriched GO terms in DEGs compared to the genome background were defined by hypergeometric test. The calculated *p*-value went through FDR correction, taking FDR ≤ 0.05 as the threshold. GO terms meeting this condition were defined as significantly enriched GO terms in DEGs. This analysis was able to recognize the main biological functions that DEGs exercise.

#### 4.6.4. Pathway Enrichment Analysis

Genes usually interact with each other to play roles in certain biological functions. Pathway-based analysis helps to further understand genes’ biological functions. KEGG is the major public pathway-related database [58]. Pathway enrichment analysis identified significantly enriched metabolic pathways or signal transduction pathways in DEGs compared with the whole genome background. The calculated *p*-value went through FDR correction, taking FDR ≤ 0.05 as a threshold. Pathways meeting this condition were defined as significantly enriched pathways in DEGs.

### 4.7. Data Analysis

The experimental data were presented as mean ± SEM. The experimental data were subjected to one-way analysis of variance (one-way AVONA) and Tukey’s test. The *t*-test, normality test, and logarithmic transformation were performed if necessary. The statistical significance was considered when *p* < 0.05. 

## 5. Conclusions

In conclusion, this study revealed BPF-induced acute embryonic and developmental toxicity in Chinese medaka. This study performed a 96-h acute toxic experiment at various concentrations and a 15-day chronic toxic exposure experiment of BPF at multiple concentrations (20, 200, and 2000 ng/L) on Chinese medaka under standard laboratory conditions. In the 96-h acute toxic exposure, the LC_50_ of BPF to Chinese medaka is 87.90 mg/L. In the 15-d developmental exposure, BPF caused a higher proportion of larvae with abnormalities. The transcriptomic analysis found 565 up-regulated and 493 down-regulated genes in the 2000 ng/L BPF group compared to the control group, and exposure to 2000 ng/L BPF seems to exert a more significant toxic effect than 20, 200 ng/L. Further analysis of GO and KEGG pathways indicated that the toxic mechanism of BPF may be related to endocrine and immunity systems. 

## Data Availability

The raw sequence data reported in this paper have been deposited in the Genome Sequence Archive (GSA) in National Genomics Data Center, under accession number: CRA010920, which is publicly accessible at https://ngdc.cncb.ac.cn/gsa (accessed on 6 May 2023).

## Supplementary Materials

The supporting information can be downloaded at: https://www.mdpi.com/article/10.3390/ijms241310898/s1.
