# Supplementary material for: Transcriptome Analysis of the Developmental Effects of Bisphenol F Exposure in Chinese Medaka (Oryzias sinensis)
Source: Int J Mol Sci. 2023 Jun 30;24(13):10898. doi: 10.3390/ijms241310898 (PMC10341846; doi:10.3390/ijms241310898)
Supplement: Supplementary file 1 [file ijms-24-10898-s001.zip › ijms-2444678-supplementary.pdf]

Supplementary Information

Transcriptome Analysis of the Developmental Effects of Bisphenol F Exposure in Chinese Medaka (*Oryzias sinensis*)

Zhiying Liang [1,†], Yafen Guo [1,†], Duan Pi [1,†], Xiang Li [1,†], Bingying Li [1], Yongsi Huang [1], Xiaohong Song [2], Ramji Kumar Bhandari [3] and Xuegeng Wang [1,*]

[1] Institute of Modern Aquaculture Science and Engineering, Guangdong-Macao Joint Laboratory for Aquaculture Breeding Development and Innovation, College of Life Sciences, South China Normal University, Guangzhou 510631, China
[2] The Guangxi Key Laboratory of Theory and Technology for Environmental Pollution Control, Guilin University of Technology, Guilin 541000, China
[3] Department of Biology, University of North Carolina Greensboro, Greensboro, NC 27412, USA
* Correspondence: wangxuegeng@scnu.edu.cn
† These authors contributed equally to this work.

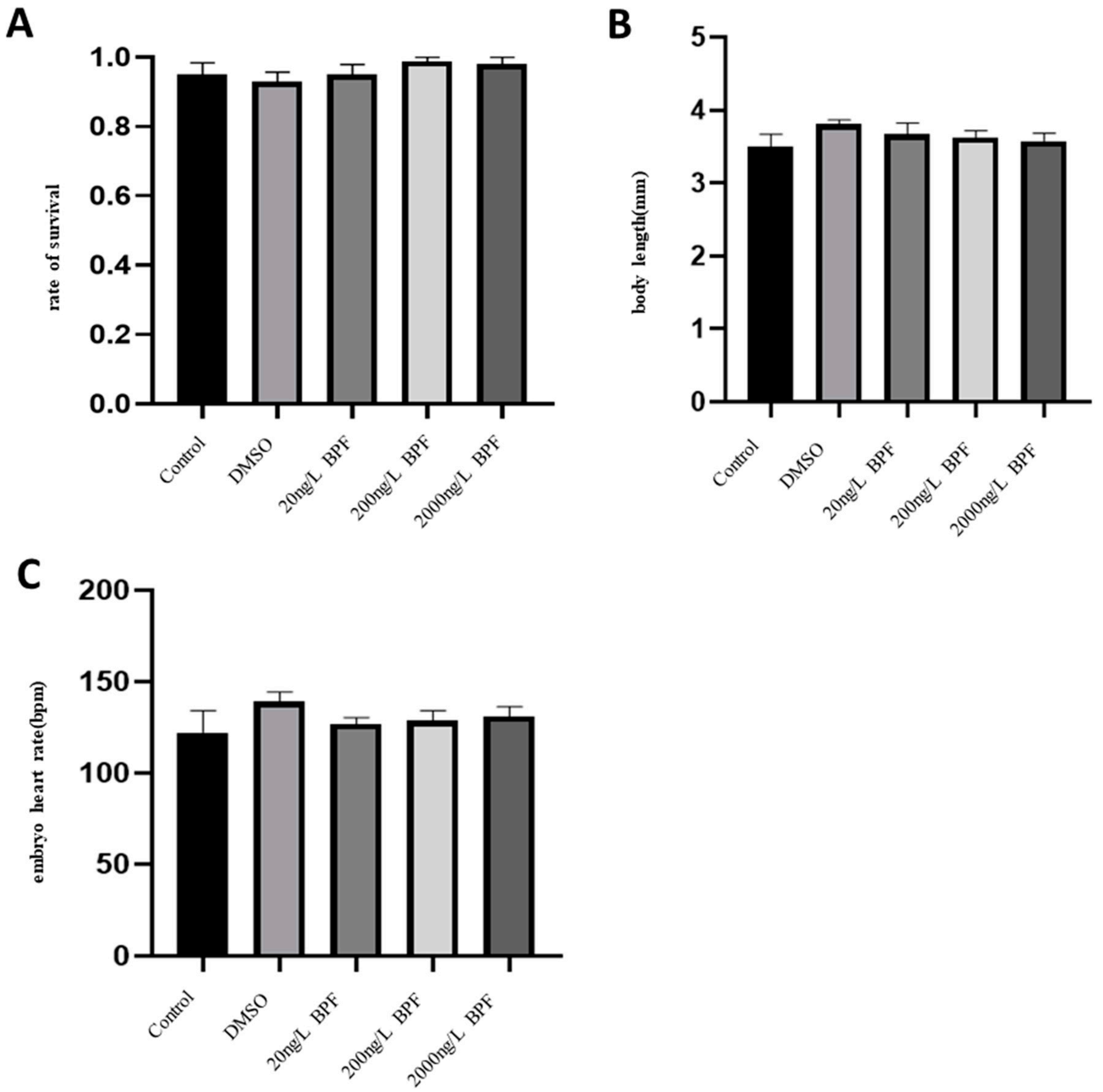

Figure S1. Results of developmental exposure. (A): Survival rates; (B): Body length (mm); (C): Embryo heart rate (bpm).

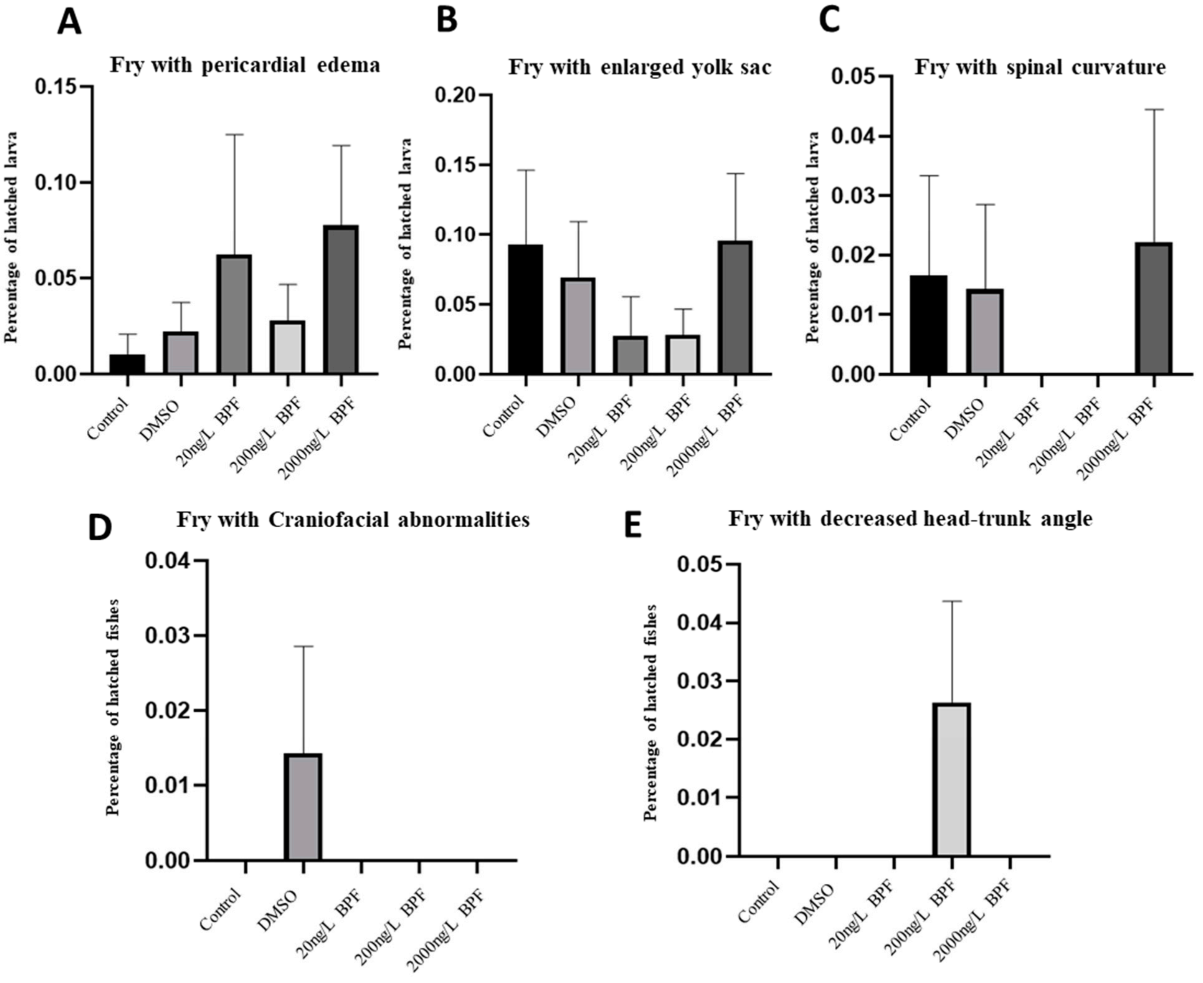

Figure S2: Percentage of larvae with abnormalities. (A): Fry with pericardial edema; (B): Fry with enlarged yolk sac; (C): Fry with spinal curvature; (D): Fry with craniofacial abnormalities; (E): Fry with decreased head-trunk angle.

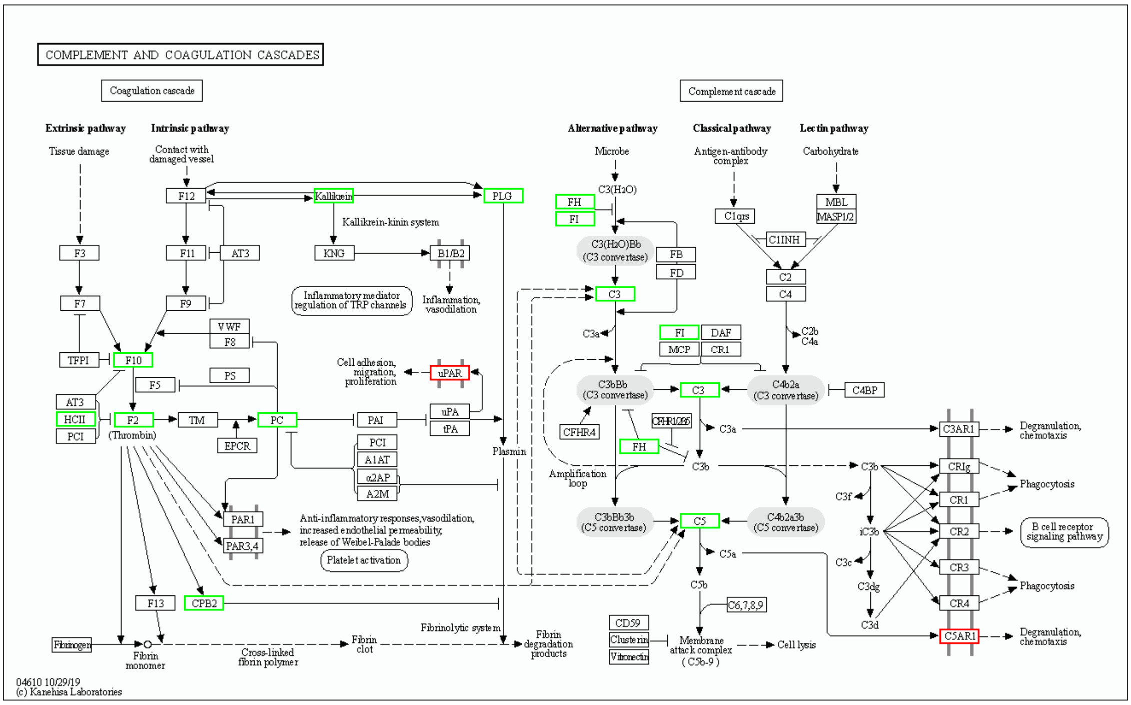

Figure S3: Changes of genes in the complement and coagulation cascades pathway. Green indicates down-regulation DEGs; red indicates up-regulation DEGs.

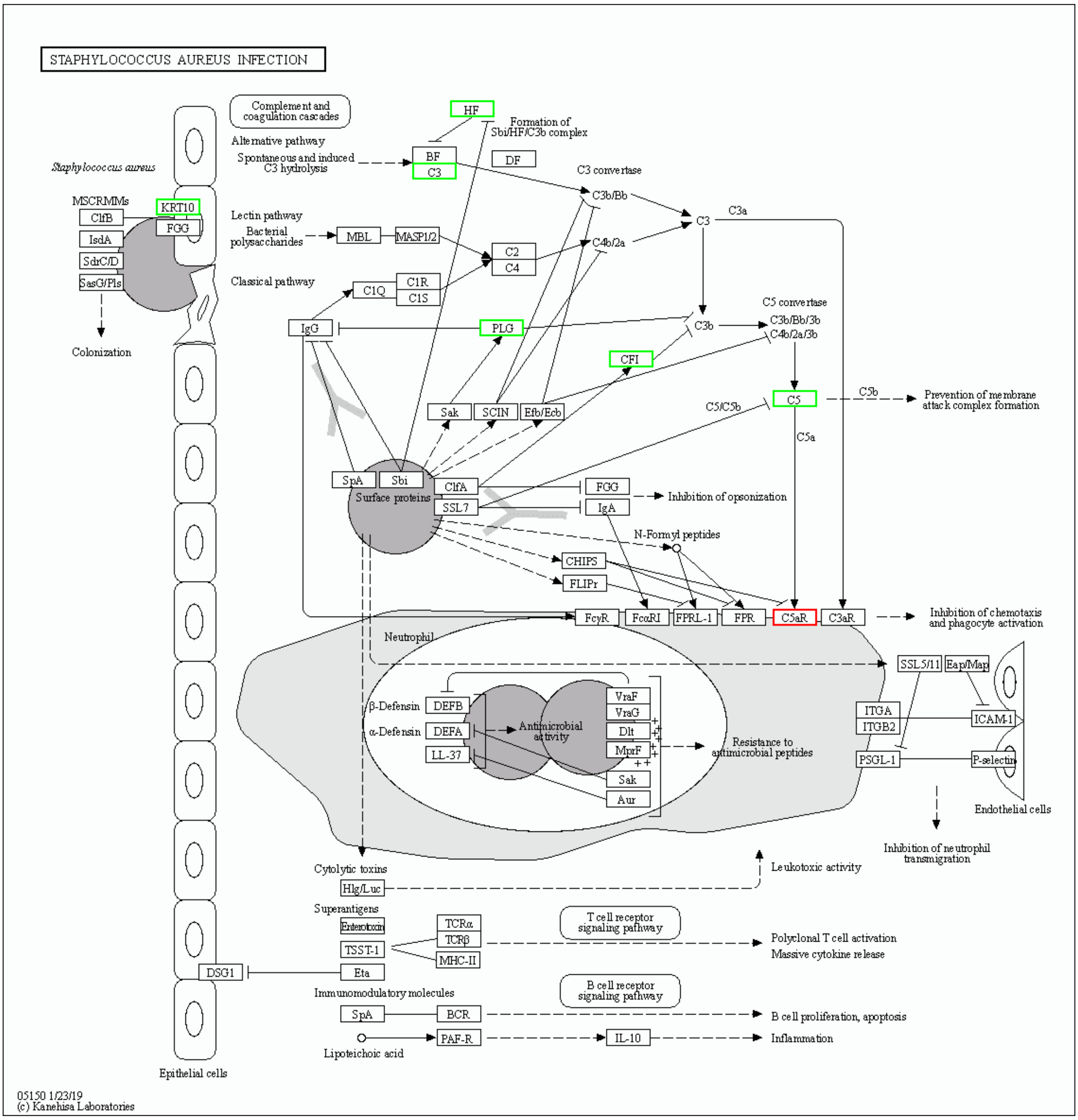

Figure S4: Staphylococcus aureus infection pathway. Green indicates down-regulation DEGs, red indicates up-regulation DEGs.

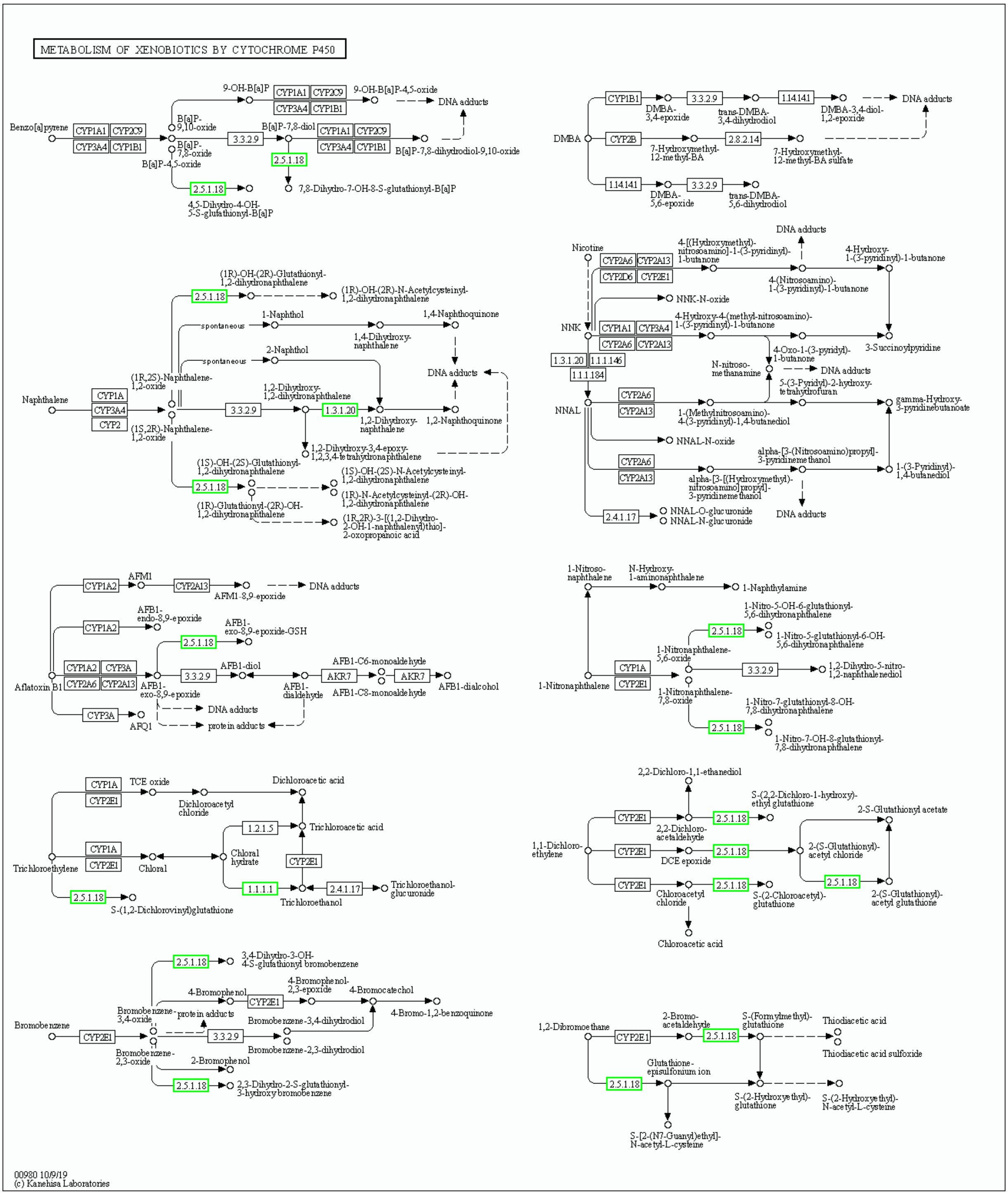

Figure S5: Metabolism of xenobiotics by cytochrome P450 pathway. Green indicates down-regulation DEGs.

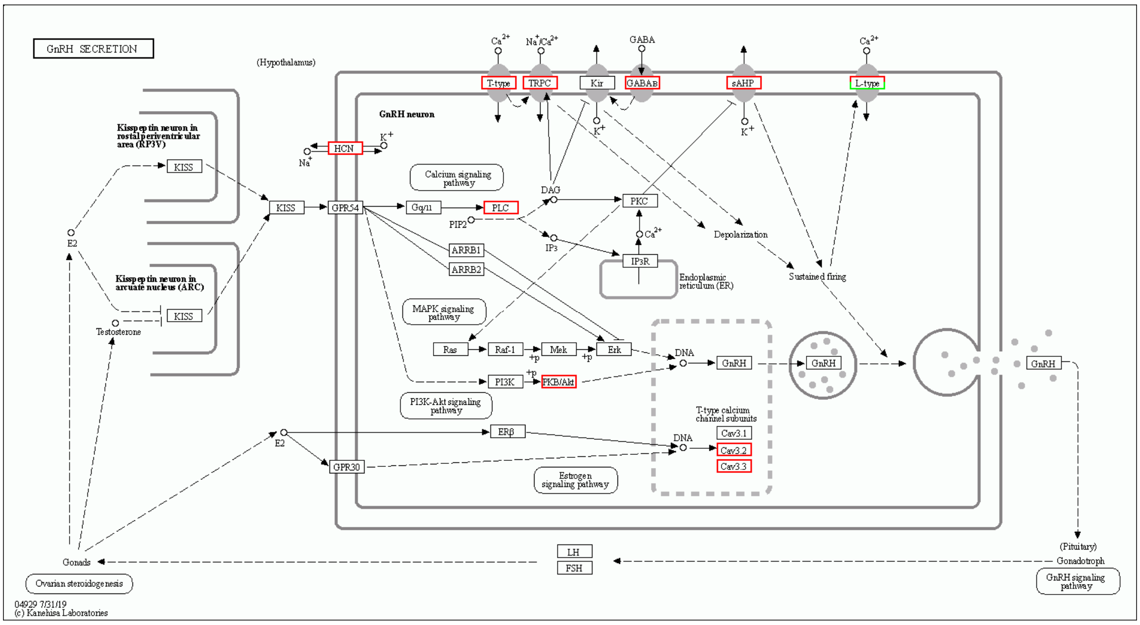

Figure S6: GnRH secretion pathway. Green indicates down-regulation DEGs; red indicates up-regulation DEGs.
